# Lifestyle Scores and Behavior Change Mindset: A Cross‐Cultural Validation Study of Simple Lifestyle Indicator Questionnaire in Farsi

**DOI:** 10.1002/hsr2.72239

**Published:** 2026-04-02

**Authors:** Amir‐Hossein Memari, Abbas Mirzapour, Keyvan Karimi, Elaheh Dehghani, Zahra Gohari Dezfuli, Minoo HasanRashedi, Fereshteh Torki, Tohid Seif Barghi

**Affiliations:** ^1^ Sports Medicine Research Center, Neuroscience Institute Tehran University of Medical Sciences Tehran Iran; ^2^ Department of Epidemiology and Biostatistics School of Public Health Tehran University of Medical Sciences Tehran Iran; ^3^ Department of Clinical Nutrition School of Nutritional Sciences and Dietetics Tehran University of Medical Sciences Tehran Iran; ^4^ Department of Nutrition School of Public Health Iran University of Medical Sciences Tehran Iran; ^5^ Department of Occupational Therapy Faculty of Rehabilitation Tehran University of Medical Sciences (TUMS) Tehran Iran

**Keywords:** behavior change, Farsi validation, lifestyle assessment tool, Simple Lifestyle Indicator Questionnaire

## Abstract

**Background and Aims:**

Examining lifestyle factors is an essential part of health promotion. The goal of this study was to translate, culturally adapt, and validate the Simple Lifestyle Indicator Questionnaire (SLIQ) for Farsi‐speaking populations. Additionally, it explored the relationship between lifestyle scores and behavior change mindset, focusing on readiness for change and personal control.

**Methods:**

A total of 91 healthy individuals were recruited and asked to complete the Farsi SLIQ, the International Physical Activity Questionnaire (IPAQ), and the Healthy Lifestyle and Personal Control Questionnaire (HLPCQ). A translation‐back translation process was used to adapt the SLIQ, ensuring accuracy in cultural and linguistic contexts. Psychometric properties, including reliability and validity, were evaluated using Cronbach's *α*, intraclass correlation coefficients (ICCs), and Spearman's rank correlations. The relationship between behavior‐change dimensions and SLIQ scores was obtained using multivariable linear regression models.

**Results:**

The Farsi SLIQ showed moderate reliability as shown by Cronbach's *α* of 0.549 and significant correlations with IPAQ (*r* = 0.250, *p* < 0.01) and HLPCQ (*β* = 0.372, *p* < 0.05). Higher SLIQ scores were linked to better adherence to healthy behaviors and increased readiness for change. Adaptation showed cultural appropriateness with slight alterations. Regression analysis highlighted a significant relationship between SLIQ scores and daily routines, dietary choices, and personal control.

**Conclusion:**

The Farsi SLIQ is a reliable and valid tool for assessing lifestyle behaviors in clinical and research settings. Because the scale is simple, it is well suited for behavior‐change interventions; our findings also show that perceived control and readiness to change are key drivers of healthy lifestyles. However, findings should be interpreted cautiously because self‐reported data, particularly on alcohol and tobacco use, may be biased.

## 1 Introduction

A healthy lifestyle is defined by the World Health Organization as “a way of living that lowers the risk of being seriously ill or dying early” [1]. Ignoring the importance of lifestyle elements such as a healthy diet and physical activity can give rise to noncommunicable diseases, specifically cardiovascular diseases and cancers [2, 3]. For example, a sedentary lifestyle linked to a diet high in processed food increases the risk of developing colorectal cancer and cardiovascular conditions like hypertension due to chronic inflammation and metabolic conditions [4, 5]. Furthermore, physical inactivity accelerates the progression of cardiovascular diseases by promoting obesity and arterial dysfunction [5, 6]. Tobacco consumption leads to over 8 million deaths a year, and second‐hand smoke causes an estimated 1.3 million deaths a year among nonsmokers [7]. Alcohol consumption, high BMI, and low levels of physical activity are also considered preventable risk factors for premature mortality and morbidity [8]. Assessing a healthy lifestyle is essential for classifying cardiovascular disease risks and developing strategies to enhance life expectancy [9, 10]. Therefore, several tools have been developed to measure an overall score of the individual's lifestyle state [11, 12, 13, 14, 15, 16]. These lifestyle assessment tools have proven useful in both research and clinical settings. They are tailored to specific populations, such as different age groups and patients with certain diseases or conditions [17, 18, 19].

Several lifestyle assessment tools have been developed that have taken factors like diet, exercise, and other lifestyle factors into account, which are necessary for recognizing individuals at risk and for preventing noncommunicable diseases [20, 21, 22, 23]. As for the importance of preventive care in recent years, lifestyle interventions have become more relevant, and lifestyle assessment tools have been useful in estimating the effects of such interventions [24, 25]. This study focuses on validating the Simple Lifestyle Indicator Questionnaire (SLIQ) in Farsi to assess lifestyle behaviors among Farsi‐speaking populations. The SLIQ has been widely used in research due to its simplicity and brevity, making it ideal for clinical and research settings where time is limited [14]. This research also explores how readiness for behavior change and personal control relate to lifestyle scores, aiming to provide a practical tool for health interventions. Although short, the SLIQ has been shown to measure each aspect of lifestyle comparably to other longer and more complex tools [14] and we decided to continue the validation of this valuable tool in this current study.

To deliver lifestyle interventions more effectively, attention to psychological concepts like personal control and behavior change techniques is of great importance [26, 27]. Strategies and techniques, based on behavior change frameworks, have been successfully used in lifestyle interventions to promote physical activity, healthy eating, and smoking cessation [28, 29].

A useful behavior change framework is provided by the transtheoretical model that can enhance the success of behavior change interventions [30]. The model has several core constructs, two of which are the stages of change and processes of change. To obtain and maintain healthy behavior, people progress through five stages of change. These stages, representing a temporal dimension, are pre‐contemplation, contemplation, preparation, action, and maintenance. The processes of change, the second dimension of the model, are defined as the behaviors and attitudes that people need to have to advance from one stage to the next. To assess the different aspects of behavior change, several tools have been developed based on the transtheoretical model. The URICA is a widely used measure designed to estimate readiness for change [31]; for example, it was used in a range of different problems like smoking, alcohol, weight management, and obesity [32, 33]. The Decisional Balance Measure, focusing on the pros and cons of weight reduction, was designed to assess decision‐making in weight control [34]. Also, S‐weight and P‐weight are two questionnaires that investigate, respectively, the stages and processes of change in weight management [35].

Behavior change models state the importance of personal control and psychological readiness in achieving and sticking to healthy behaviors [36]. Interventions that target different dimensions of individual lifestyles find this mindset shift relevant [36]. However, most of the tools either assess readiness for change or lifestyle factors, and few studies have investigated the relationship between these two concepts.

This study investigates the possible relations between the mindset of change, including the processes and stages of change, personal control, and the state of the current lifestyle. As there is a need for more brief lifestyle assessment tools in Farsi, another objective of this study was to translate and culturally adapt the SLIQ [14] into Farsi and assess its psychometric properties in a new sample of the population.

## 2 Methods and Materials

### 2.1 Participants

The study included 91 healthy participants and they were recruited through flyers, call and in person invitations from the research center and received assurances of the confidentiality and privacy of their data and signed a consent form in May 2023. Participants completed the Farsi‐translated SLIQ, as well as the International Physical Activity Questionnaire (IPAQ) and Healthy Lifestyle and Personal Control Questionnaire (HLPCQ). To ensure accuracy, the SLIQ was translated into Farsi using a back‐translation method. We evaluated the reliability of the tool using Cronbach's *α* and the intraclass correlation coefficient (ICC), and we tested its validity by comparing SLIQ scores with IPAQ and HLPCQ results.

### 2.2 Measures

#### 2.2.1 Sociodemographic Data

The clinical and sociodemographic data were gathered using an online questionnaire. The sociodemographic information that was gathered included age, gender, height, weight, education level, work status, reason for referring to the clinic, and marital status.

#### 2.2.2 Simple Lifestyle Indicator

The SLIQ was utilized to measure lifestyle [14]. SLIQ has 12 items total, divided into 5 categories: 3 of them are related to diet, 3 to exercise, 3 to alcohol intake, 2 to tobacco use, and 1 to life stress. In addition, its scale ranging from 0 to 5 for diet, 0–4, 0–8, and 0–12 for exercise, 0–2 for smoking, 1–6 for life stress, and with no range for alcohol consumption is used to evaluate each item in each dimension independently. Nevertheless, a single category score per dimension, ranging from 0 to 2, is assigned to these scores once they are combined. Each dimension has a different set of criteria used to determine this score. For the diet dimension, a score of 0 is given if the total direct score falls between 0 and 5, a score of 1 falls between 6 and 10, and a score of 2 falls between 11 and 15. The exercise category will be 0 if the person only exercises lightly, a 1 if they exercise moderately, and a 2 if they routinely exercise vigorously. Regarding alcohol consumption, a raw score of 0 is given for drinking 14 or more drinks, a raw score of 1 for drinking 8–13 drinks, and a score of 2 for drinking 0–7 drinks. Regarding tobacco use, a participant receives a score of 0 if they smoke currently, a score of 1 for those who have smoked in the past, and a score of 2 if they have never smoked. Lastly, when it comes to life stress, a 0 is given for values of 1 or 2, a 1 for values of 3 or 4, and a 2 for values of 5 or 6. The sum of the scores assigned to each of the five dimensions of the SLIQ questionnaire, which range from 0 (“unhealthy”) to 10 (“healthy”), is the overall score. Individuals' lifestyles are classified as “unhealthy” if their SLIQ score falls between 0 and 4, “partially healthy” if it falls between 5 and 7, and “healthy” if it falls between 8 and 10 on the SLIQ questionnaire [14].

#### 2.2.3 The Healthy Lifestyle and Personal Control (HLPCQ)

The rating of participants by the HLPCQ questionnaire was done based on their frequency in adopting 26 positive‐biased lifestyle questions on a Likert‐type scale (1 = *never or rarely*, 2 = *sometimes*, 3 = *often*, and 4 = *always*) which 12 of the items are linked to dietary harm avoidance and healthy food choices (DHC), 8 to the daily routine (DR), 2 to organized physical activity (OPA), and 4 to social and mental balance (SMB). High scores show a person's empowerment over their health [37]. Greek formatting and validation of the HLPCQ questionnaire were done by Darviri et al. [15].

#### 2.2.4 Physical Activity

The short form of the IPAQ consists of seven items [38], and its application is physical activity (PA) measurement. IPAQ asks about walking and sitting durations as well as the frequency, duration, and intensity of exercise over the previous 7 days. This questionnaire specifically assesses three aspects of physical activity: frequency (days per week), length (minutes per day), and intensity (mild, moderate, or intense). Physical Activity Level is classified as “Inactive,” “Minimally active,” or “Active” based on the findings [38].

#### 2.2.5 Stages and Processes of Weight Management (S‐Weight and P‐Weight)

These questionnaires have been developed by experts in the fields of obesity and transtheoretical models, respectively, and were validated in Farsi [39] and then used to evaluate the stages and processes of change for weight management [35, 40]. According to the Delphi method's validation, both surveys have a high level of consensus (around 80%). In the P‐weight, participants were given a 5‐point Likert scale, with 1 signifying strong disagreement and 5 denoting strong agreement, to indicate how much participants agreed or disagreed with items regarding their weight and eating habits. The P‐weight evaluates four transformation processes related to weight management: environmental restructuring, emotional reevaluation, weight management behaviors, and weight consequences assessment.

### 2.3 Cross‐Cultural Adaptation

The process of cross‐cultural adaptation adhered to the guidelines for transcultural adaptation of self‐reported questionnaires [33]. Initially, permission was obtained from the original authors. A straight conceptual translation from the English original version of the SLIQ questionnaire into Farsi was carried out as part of the translation‐back translation and adaptation process. A blind back‐translation method was then used to translate the initial Farsi version into English by a bilingual translator who was not familiar with the original SLIQ instrument. The final phase involved comparing the back‐translated translation with the original text by a third, objective translator to ensure linguistic equivalence while considering cultural differences. Minor adjustments were made in this case, like eliminating uncommon and little‐known activities (like volunteering) and adding comparable things where certain activities are unique. Two new questions about two high‐consumption food groups (dairy and oils) and particular adjustments to the alcohol measurement (such as replacing ounces with cubic centimeters) were added. Finally, the cultural and linguistic accuracy of the translated questionnaire was guaranteed by a committee made up of six Iranian specialists in lifestyle assessment. Appendix S1A contains the Farsi version of the SLIQ.

### 2.4 Assessment Procedure

The Ethical Principles for Medical Research Involving Human Subjects [41] were followed in the approval of the study by the Tehran University of Medical Sciences (IR.TUMS.NI.REC.1398.025). An information sheet regarding the study informed consent for participation, and the many assessment instruments collected in the project were all included in the anonymous and confidential online assessment procedure developed to administer the questionnaires. All participants (*n* = 91) completed the entire set of questionnaires contained in the evaluation protocol, which comprised the SLIQ, IPAQ, HLPCQ, P‐weight, and S‐weight.

### 2.5 Statistical Analysis

Data were entered in Stata version 18 to define variables and check for unusual values. To assess the reliability of the SLIQ questionnaire, Cronbach's *α* was used, and the interclass correlation coefficient with their corresponding 95% confidence intervals were calculated to evaluate inter‐item reliability between items in each dimension. To determine the convergent validity of the SLIQ, the bivariate Spearman's rank correlation between the scores of the SLIQ and the IPAQ (physical activity questionnaire) was used.

The frequency (numerator) and percentage of categorical parameters were summarized in three lifestyle subgroups based on the SLIQ scores, namely, unhealthy, partially healthy, and healthy. According to normal or abnormal distribution, numerical variables were also described as mean along with standard deviation or median (interquartile range). One‐way analysis of variance (ANOVA) and the Kruskal–Wallis tests were used to compare the mean values of P‐weight, HLPCQ (and their specific dimensions), and the median of S‐weight in the lifestyle subgroups. Linear regression was performed to examine the relationship between SLIQ scores as dependent variables (DV) and HLPCQ total scores (and specific dimensions) in five quantiles as independent variables (IV). In addition, a multivariable linear regression with age, sex, BMI, and marital status as potential confounders was performed to estimate the adjusted association between DV and IV. The regression results were presented as a *β* coefficient with a 95% confidence interval.

In addition, the trend of changes in the SLIQ score across the BMI quintile, physical activity quintile, and stage of weight management was depicted in a line graph. In all analyses, two‐sided and *p* < 0.05 was interpreted as statistically significant.

## 3 Results

The mean age of the participants was 39 ± 16.4 years old. Of these, 64.8% had a history of diabetes, hypertension, or cardiovascular diseases, 34.1% of the participants were male, a normal BMI was reported by 49.5% of individuals, and according to IPAQ, 46.1% of patients led an active lifestyle. Additionally, 53.8% were married, and the *p* value of 0.008 indicates a significant difference in marital status across the groups. The mean age between different lifestyle groups is significantly different, which shows healthier individuals tend to be older. Table 1 presents a description of the other participant characteristics.

**Table 1:** The characteristics of the study population by lifestyle categories (*n* = 91).

Parameter	Lifestyle categories			*p*	Total population
	Unhealthy (*N* = 10)	Partially healthy (*N* = 46)	Healthy (*N* = 35)		
Age mean (51)	28 (7.4)	37 (15.6)	46 (16.7)	0.002	39 (16.4)
Male *N* (%)	3 (30)	17 (36.9)	11 (31.4)	0.838	31 (34.1)
Married *N* (%)	4 (40)	19 (41.3)	26 (74.3)	0.008	49 (53.8)
Disease history *N* (%)	7 (70)	26 (56.5)	26 (74.3)	0.236	59 (64.8)
*BMI categories*					
Normal *N* (%)	3 (30)	23 (50.0)	19 (54.3)	0.124	45 (49.5)
Overweight *N* (%)	3 (30)	19 (41.3)	11 (31.4)		33 (36.3)
Obese *N* (%)	4 (40)	4 (8.7)	5 (14.3)		13 (14.3)
*Physical activity (IPAQ)*					
Inactive *N* (%)	4 (40)	15 (32.6)	7 (20.0)	0.580	26 (28.6)
Minimal active *N* (%)	3 (30)	10 (21.7)	10 (28.6)		23 (25.3)
Active *N* (%)	3 (30)	21 (45.6)	18 (51.4)		42 (46.1)

Abbreviations: BMI, body mass index; IPAQ, International Physical Activity Questionnaire; *N*, Number of frequencies; SD, standard deviation.

### 3.1 Cross‐Cultural Adaptation

Cultural adaptations were made to reflect local dietary habits. Adjustments were made to the methods of measuring alcohol consumption to better align with Iranian cultural norms. The adaptation process showed no difficulties during the review of the translations or back translations. There were only minimal changes in the wording of Items 3, 7, and 9. However, following expert recommendations, all experts confirmed the statements from translations and confirmed the final scale.

### 3.2 Reliability and Convergent Validity

The Farsi SLIQ demonstrated moderate reliability (Cronbach's *α* = 0.549) and showed a significant positive correlation with physical activity (IPAQ: *r* = 0.250, *p* < 0.01), indicating that it effectively assesses lifestyle behaviors and is meaningfully related to physical activity levels. The ICC for multi‐question measures in the SLIQ questionnaire was 0.684 (*p* < 0.001) for the total diet score, 0.496 (*p* < 0.001) for physical activity, 0.601 (*p* < 0.001) for smoking and 0.586 (*p* < 0.001) for alcohol questions, respectively. According to the literature, this indicates good to excellent agreement. For other modules, including life stress, smoking, and alcohol consumption, ICC was not calculated due to the single item. As for the convergent validity, the correlation of the SLIQ questionnaire with the IPAQ questionnaire for physical activity was assessed (*r* = 0.250 *p* < 0.01). Overall, the results suggest that the SLIQ can be a reliable tool for assessing lifestyle behaviors in clinical and research settings.

### 3.3 Relationship Between SLIQ Scores and Other Instruments

#### 3.3.1 Healthy Lifestyle and Personal Control (HLPCQ)

There is a significant relationship between the SLIQ scores and the HLPCQ scores. The median HLPCQ score increased significantly across SLIQ categories, from 61.6 in the “unhealthy” group to 74.6 in the “healthy” group (*p* < 0.001). In addition, notable increases were seen in specific HLPCQ subscales, such as Dietary Healthy Choice (DHC) (*p* = 0.027), Dietary Harm Avoidance (DHA) (*p* = 0.003), Daily Routine (DR) (*p* = 0.004), and Organized Physical Exercise (*p* = 0.014), indicating that higher SLIQ scores are associated with more personal control over lifestyle choices.

To explore the relationship between HLPCQ and SLIQ with and without alcohol consumption (AC), multivariable linear regression was performed, and both crude and adjusted models were evaluated. The crude model revealed a significant association between HLPCQ score and SLIQ with AC (*β* = 0.411, 95% CI = 0.182–0.641) and without AC (*β* = 0.425, 95% CI = 0.210–0.640). After adjusting for age, sex, marital status, and BMI, the associations remained significant but slightly decreased (*β* = 0.372, 95% CI = 0.139–0.605 for SLIQ with AC; *β* = 0.408, 95% CI = 0.188–0.627 for SLIQ without AC). Among the HLPCQ subscales, the Daily Routine (DR) subscale showed the most significant association with SLIQ without AC (*β* = 0.462, 95% CI = 0.245–0.679). These findings suggest that improvements in healthy lifestyle practices, particularly in daily routine, are associated with better SLIQ scores, regardless of alcohol consumption (Table 3).

#### 3.3.2 Stages and Processes of Weight Management (P‐Weight and S‐Weight)

The results of S‐weight showed a significant association with SLIQ scores (*p* = 0.002), with median scores rising from 2 in the “unhealthy” group to 5 in the “healthy” group. However, no significant differences were found in P‐weight scores or subscales of this tool across the SLIQ categories, as indicated by their *p* values (*p* > 0.05) (Table 2).

**Table 2:** Summary of P‐weight and S‐weight scores by SLIQ categories.

Parameter	Unhealthy (*N* = 10)	Partially healthy (*N* = 46)	Healthy (*N* = 35)	*p*
P‐weight score; median (IQR)	114.5 (46)	111.5 (22)	117 (32)	0.354
EmR median; (IQR)	41 (8)	42.5 (13)	42 (11)	0.871
WCE; mean (51)	17.8 (6.3)	15.5 (5.2)	15.4 (5.1)	0.441
SR; median (IQR)	17 (6)	17.6 (6)	17 (6)	0.911
WMA; mean (51)	27.5 (10.5)	29.5 (7.5)	32.9 (8.3)	0.088
S‐weight; median (IQR)	2 (3)	3 (2)	5 (3)	0.002

Abbreviations: DHA, Dietary Harm Avoidance; DHC, Dietary Healthy Choice; DR, Daily Routine; EmR, emotional reevaluation; HLPCQ, Healthy Lifestyle and Personal Control Questionnaire; IQR, interquartile range; *N*, number of frequencies; OPE, Organized Physical Exercise; P‐weight, process of weight management; S‐weight, stage of weight, SD, standard deviation; SMB, social and mental balance; SR, supporting relationship; WCE, weight consequences evaluation; WMA, weight management actions.

**Table 3:** Association between HLPCQ and SLIQ.

Independent^a^ Variable	SLIQ with AC		SLIQ without AC	
	Crude model *β* (95% CI)	Adjusted^b^ model *β* (95% CI)	Crude model *β* (95% CI)	Adjusted model *β* (95% CI)
HLPCQ	0.411 (0.182–0.641)	0.372 (0.139–0.605)	0.425 (0.210–0.640)	0.408 (0.188–0.627)
DHC	0.455 (0.219–0.691)	0.403 (0.166–0.640)	0.455 (0.234–0.677)	0.425 (0.201–0.649)
DHA	0.320 (0.088–0.553)	0.298 (0.066–0.529)	0.358 (0.140–0.575)	0.350 (0.132–0.568)
DR	0.452 (0.221–0.684)	0.447 (0.220–0.673)	0.462 (0.245–0.679)	0.465 (0.251–0.679)
OPE	0.389 (0.162–0.617)	0.374 (0.149–0.600)	0.408 (0.195–0.621)	0.412 (0.201–0.624)
SMB	0.189 (−0.057–0.436)	0.193 (−0.49–0.435)	0.211 (−0.022–0.444)	0.224 (−0.007–0.455)

Abbreviations: *β*, regression coefficient; AC, alcohol consumption; CI, confidence interval; DHA, Dietary Harm avoidance; DHC, Dietary Healthy Choice; DR, Daily Routine; HLPCQ, Healthy Lifestyle and Personal Control Questionnaire; OPE, Organized Physical Exercise; SLIQ, Simple Lifestyle Indicator Questionnaire; SMB, social and mental balance.

a Independent variables were defined in five quantiles.

b Adjusted for age, sex, marital status, and BMI.

#### 3.3.3 Body Mass Index (BMI)

**Figure 1:**
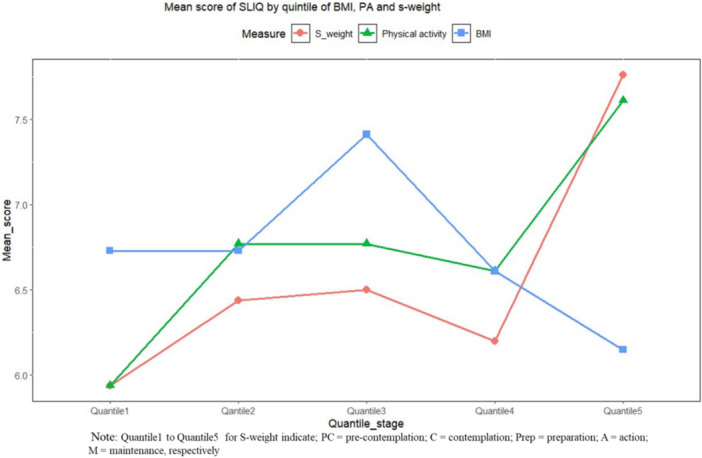
The mean change of the Simple Lifestyle Indicator Questionnaire (SLIQ) score across body mass index (BMI), physical activity, and stage of weight (S‐weight). This figure shows the trend in the mean change of Simple Lifestyle Indicator Questionnaire (SLIQ) scores across different body mass index (BMI) categories, physical activity levels, and stages of weight management (S‐weight).

The relationship between BMI and SLIQ scores showed a complex trend. SLIQ scores remained stable across the first three BMI quintiles, peaked sharply in the fourth quintile, and then declined significantly in the fifth quintile. This suggests that moderate BMI levels are associated with better adherence to a healthy lifestyle, while extremely high BMI correlates with poorer lifestyle adherence (Figure 1).

## 4 Discussion

The main goal of this study was to translate and validate one of the most crucial tools for clinical and research use, the SLIQ, in Farsi. It also explores the relationship between lifestyle scores and behavior change mindset in the Iranian population. The Farsi version of the SLIQ provides a quick and accessible way to assess lifestyle characteristics, which can be especially valuable in clinical settings where time constraints may prevent patients from doing comprehensive assessments. This validation study is important since it provides a culturally relevant tool for assessing the Iranian population's lifestyle, which potentially may lead to designing and implementing interventions aimed at health behavior change. This study highlights the crucial role of psychological factors, including personal control and the readiness for behavior change, in the promotion of a healthy lifestyle.

### 4.1 SLIQ Validation

The cross‐cultural adaptation of SLIQ was carried out to ensure both linguistic and cultural relevance. The process involved translation, translation‐back, and expert committee assessment with minor adjustments to reflect Iranian dietary habits and lower prevalence of alcohol use; for instance, some foods were replaced by more high‐consumption food groups like dairy and oils. These modifications did not alter the core components of the questionnaire, preserving its ability to measure key lifestyle dimensions effectively. Our results indicate that the Farsi version of SLIQ has moderate reliability and adequate convergent validity as evidenced by its correlation with the IPAQ for physical activity. In this study, Cronbach's *α* of 0.549 was calculated for the Farsi version of SLIQ, which demonstrates acceptable internal consistency. Future studies can consider enhancing certain items to improve internal consistency.

Ultimately, the evidence indicates that the adapted version of the SLIQ exhibits equivalent psychometric properties, thereby affirming its alignment with the original measure and the Spanish validation of the test [42]. In our study, the diet and physical activity dimensions of the Farsi SLIQ showed significant correlations with the HLPCQ and IPAQ, with correlation coefficients of *r* = 0.466 and *r* = 0.490, respectively. Although these correlations were slightly lower than those found in the original study, they remained statistically significant, supporting the validity of the Farsi version of the SLIQ for assessing key lifestyle dimensions.

Additionally, utilizing the behavior change framework in our study, where psychological readiness and personal control are considered important factors for maintaining long‐term lifestyle changes [36], could support a more holistic perspective in lifestyle interventions, ultimately contributing to improved public health outcomes.

There was a nonlinear pattern in the relationship between SLIQ scores and BMI. Those individuals with extremely high or low BMI show less adherence to healthy lifestyle behaviors and individuals with moderate BMI commit to healthy lifestyle behaviors. Similar trends have been recognized in other studies in which healthier lifestyle choices with moderate BMIs likely because of better dietary habits and better physical capacity [43]. However, in our study, SLIQ integrates multiple lifestyle factors, contrary to instruments that solely focus on one particular domain, such as nutrition or physical activity [44].

### 4.2 The Effect of Mindset on Healthy Lifestyle Choices

According to the results, individuals with higher SLIQ scores tend to be further along in the stages of change and have more influence on personal lifestyle decisions. This is consistent with research by Prochaska and DiClemente, which found that long‐term gains in health behaviors, such as quitting smoking and making dietary adjustments, were linked to readiness for change [36]. These results provide credence to the notion that encouraging long‐term healthy living behaviors requires a positive behavior change mindset.

Similar findings have been found in studies that assess other health behaviors like physical activity and dietary adherence. Self‐efficacy and personal control, for instance, are predictive of continued participation in health‐promoting activities, indicating that people who feel more in control of their routines and dietary choices are more likely to experience improved health outcomes [45]. Studies using the HLPCQ have also found that higher scores on daily routine and dietary health choices subscales are strongly associated with adherence to a healthy lifestyle [46]. The transtheoretical model of behavior change aligns with this finding, indicating that higher lifestyle scores are associated with improved health outcomes and a stronger internal motivation for healthy behavior.

However, there are some contradictions in the literature. In a study by Anderson and Fowers, while personal control was positively associated with some lifestyle factors, the relationship was not significant for physical activity, suggesting that factors beyond individual control may influence engagement in certain health behaviors [47]. Cultural or environmental differences may be the reason; for instance, personal control may have a smaller impact on lifestyle choices in settings where access to exercise facilities or nutritious foods is limited. In summary, what our study contributes to the literature is the significance of mindset in maintaining a healthy lifestyle. It can be interpreted from the significant association between SLIQ scores and the HLPCQ subscales, particularly in areas such as daily routine and dietary health choices.

### 4.3 Mechanisms

In order to explain possible underlying mechanisms, we should mention psychological, behavioral, and clinical perspectives. The concept of habit formation is one underlying psychological mechanism that could explain this finding. Maintaining regular daily routines and structured physical activities can cause automaticity. As a result, sustaining healthy habits will no longer need constant motivation or decision‐making [48]. This automaticity can be particularly beneficial in the context of behavior change models like the transtheoretical model, as it helps individuals move from the contemplation and preparation stages into action and maintenance by reducing cognitive load and increasing consistency in healthy habits [49].

From a behavioral perspective, self‐efficacy and personal control can improve adherence to health behaviors. Thus, as individuals successfully integrate structured activities into their routines, they build a sense of accomplishment that promotes health behavior change [50]. In terms of clinical and biological mechanisms, regular exercise has been demonstrated to provide favorable physiological changes, including enhanced cardiovascular health, decreased inflammation, and improved metabolic function [51]. These biological benefits can increase physical well‐being and energy levels, making it easier for individuals to engage in other healthy behaviors, such as maintaining a balanced diet and managing stress.

This suggests that structured approaches to lifestyle modification, guided by behavior change models, may be particularly effective in helping individuals progress through the stages of change and adopt more healthy behaviors. By integrating psychological concepts like personal control and readiness for change, health interventions can be more tailored to individual needs, ultimately improving the efficacy of lifestyle interventions.

### 4.4 Implication and Limitation

The Farsi version of the SLIQ provides a valuable tool for research and clinical use to assess and track lifestyle behaviors in Iranian populations. Because of its briefness, it would be particularly suitable for clinicians, especially for clinicians working in busy healthcare settings where time is often limited. The SLIQ can help clinicians quickly assess patients' lifestyle behaviors, identify areas for improvement, and tailor interventions accordingly.

Furthermore, the relationship between SLIQ scores and behavior change mindset underlines the importance of integrating psychological concepts like personal control into lifestyle interventions. By integrating these psychological concepts into lifestyle interventions, clinicians and public health professionals can enhance the effectiveness of their strategies, making them more individualized and impactful.

Despite the promising findings, this study has several limitations. The use of self‐reported data could introduce bias, particularly in sensitive areas such as tobacco and alcohol use. These behaviors are often subject to social desirability bias, where participants may underreport unhealthy behaviors to present themselves in a more favorable light. Additionally, cultural factors may influence the willingness of participants to disclose information about behaviors that are socially stigmatized or considered taboo in certain contexts, such as alcohol consumption in some communities. To mitigate these biases, future studies should consider using objective measures where possible, such as biomarkers for alcohol and tobacco use, or anonymous data collection methods that reduce the social pressure to conform. Future studies with larger, more diverse samples and objective measures of lifestyle behaviors are needed to further validate the Farsi version of the SLIQ. Moreover, longitudinal studies are needed to assess the stability of SLIQ scores over time and to examine the long‐term effects of behavior change interventions on lifestyle scores.

## 5 Conclusion

This study underscores the strong association between lifestyle and well‐being, emphasizing the role of behavior change in promoting healthier living. The Farsi version of the SLIQ has proven to be a valid and reliable tool for quickly assessing lifestyle behaviors for both clinical practice and research. By incorporating psychological aspects of behavior change, this tool can help guide interventions aimed at fostering sustainable healthy habits, ultimately contributing to the prevention of chronic diseases and the enhancement of quality of life. The findings also suggest that individuals who tend to achieve better lifestyle scores, particularly those who are more engaged in daily routines and organized physical activities, tend to have a mindset geared toward behavior change.

## Author Contributions

All authors contributed to the study conception and design. Abbas Mirzapour, Keyvan Karimi, Elaheh Dehghani, Minoo HasanRashedi, and Zahra Gohari Dezfuli performed data collection and analysis. Keyvan Karimi, Elaheh Dehghani, and Fereshteh Torki prepared the first draft of the manuscript, and all authors provided feedback on previous versions. All authors have read and approved the final version of the manuscript. Fereshteh Torki had full access to all of the data in this study and takes complete responsibility for the integrity of the data and the accuracy of the data analysis.

## Funding

The authors received no specific funding for this work.

## Disclosure

The lead author Amir‐Hossein Memari affirms that this manuscript is an honest, accurate, and transparent account of the study being reported; that no important aspects of the study have been omitted; and that any discrepancies from the study as planned (and, if relevant, registered) have been explained.

## Ethics Statement

This study was conducted in accordance with the ethical principles outlined in the Declaration of Helsinki. Ethical approval was granted by the Tehran University of Medical Sciences (IR.TUMS.NI.REC.1398.025).

## Consent

All participants provided written informed consent before participating in the study.

## Conflicts of Interest

The authors declare no conflicts of interest.

## Supporting information

Appendix 1.

## Data Availability

The data set generated and analyzed during the current study is available from the corresponding author upon reasonable request.
