# Supplementary material for: Lifestyle Scores and Behavior Change Mindset: A Cross‐Cultural Validation Study of Simple Lifestyle Indicator Questionnaire in Farsi
Source: Health Sci Rep. 2026 Apr 2;9(4):e72239. doi: 10.1002/hsr2.72239 (PMC13051919; doi:10.1002/hsr2.72239)
Supplement: Supplementary file 1 — Appendix 1. [file HSR2-9-e72239-s001.pdf]

۱. **فعالیت بدنی:** برای پاسخ به سوالات این بخش لطفا مشخص کنید که در طول هفته، چند مرتبه حداقل به مدت ۳۰ دقیقه یا بیشتر در فعالیت‌های زیر شرکت می‌کنید.

·فعالیت سبک مانند:

·نگهداری از گل و گیاه خانگی و آپارتمانی (آبیاری گیاه و باغبانی سبک)

·کارهای سبک خانه (مثل تی کشیدن، گردگیری، کشیدن جارو برقی، آشپزی و شستن ظروف)

·آهسته قدم زدن (مانند قدم زدن با حیوان خانگی)

·بولینگ، ماهی‌گیری، نجاری، نواختن ساز

- ۱ بار در هفته
- ۲ بار در هفته
- ۳ بار در هفته
- ۴ بار در هفته
- ۵ بار در هفته
- ۶ بار در هفته
- ۷ بار در هفته
- ۸ بار در هفته
- فعالیت های ذکر شده را اصلا انجام نمیدهم

۲. **فعالیت متوسط**، فعالیتی که در حین انجام آن می‌توانید یک جمله کامل صحبت کنید اما نمی‌توانید آواز بخوانید؛ مانند:

·پیاده‌روی تند

·دوچرخه‌سواری با شدت متوسط، اسکیت‌سواری، شنا، کوه‌گشت

·باغبانی (جمع کردن برگ‌ها با چنگک یا جارو، کندن علف‌های هرز، حفر چاله)

·کلاس‌های ورزشی با شدت متوسط مثل یوگا، رقص، تای‌چی، پیلاتس

- ۱ بار در هفته
- ۲ بار در هفته
- ۳ بار در هفته
- ۴ بار در هفته
- ۵ بار در هفته
- ۶ بار در هفته
- ۷ بار در هفته
- ۸ بار در هفته
- فعالیت های ذکر شده را اصلا انجام نمیدهم

۳. **فعالیت شدید**، فعالیتی که در حین انجام آن نمی‌توانید یک جمله کامل صحبت کنید؛ مانند:

·دویدن، دوچرخه‌سواری با شدت بالا، کوه‌نوردی، شنای طول استخر، ورزش ایروبیک(زومبا، ایروجیم، کراس فیت)

·فعالیت های سنگین حیاط خانه مثل باغبانی سنگین و غیره

·تمرین با وزنه (مثل بدن‌سازی)

·فوتبال، بسکتبال یا سایر ورزش‌های تیمی

- ۱ بار در هفته
- ۲ بار در هفته
- ۳ بار در هفته
- ۴ بار در هفته
- ۵ بار در هفته
- ۶ بار در هفته
- ۷ بار در هفته
- ۸ بار در هفته

- فعالیت های ذکر شده را اصلا انجام نمیدهم

۴. آیا تا به حال نوشیدنی الکلی مصرف کرده اید؟

- بله
- خیر

۵. آیا مصرف روزانه‌ی نوشیدنی های الکلی دارید؟

- بله
- خیر

۶. **مصرف الکل**: لطفا مشخص کنید که به طور میانگین در یک هفته چند واحد از نوشیدنی‌های الکلی زیر مصرف میکنید.

آبجو (هر واحد مساوی ۳۰۰-۳۶۰ سی‌سی یا یک بطری)

۷. شراب (هر واحد مساوی ۹۰-۱۵۰ سی‌سی یا یک فنجان)

۸. سایر نوشیدنی الکلی با درصد بالاتر الکل (برندی، جین، رام، ویسکی و غیره) (هر واحد ۳۰-۴۵ سی‌سی یا یک شات)

۹. **مصرف دخانیات:**

لطفا به سوالات زیر درباره مصرف دخانیات حاوی تنباکو(سیگار، قلیان، سیگار الکترونیک و پیپ) پاسخ دهید:

آیا در گذشته مصرف سیگار یا قلیان داشته اید؟

- بله مصرف روزانه سیگار و یا سایر دخانیات داشتم
- بله مصرف سیگار و یا سایر دخانیات داشتم اما کمتر از روزانه
- خیر مصرف سیگار و یا سایر دخانیات نداشتم

۱۰. در حال حاضر، چقدر مصرف سیگار و قلیان (و یا سایر دخانیات) دارید؟

- هر روز سیگارو یا سایر دخانیات میکشم
- مصرف سیگار و یا سایر دخانیات دارم اما کمتر از هر روز

- اصلا سیگار و یا سایر دخانیات نمی کشم

۱۲. **استرس زندگی:** برای پاسخ به این سوال، لطفا عددی که احساس می‌کنید بیشترین تطابق را با سطح استرس در زندگی روزانه شما دارد،علامت بزنید.

استرس زیاد: ۱

استرس کم: ۶

- ۱
- ۲
- ۳
- ۴
- ۵
- ۶
